# Ellagic Acid as a Promising Antifungal Agent: A Review of Mechanisms, Synergy, and Formulation Strategies

**DOI:** 10.3390/antibiotics15010072

**Published:** 2026-01-09

**Authors:** Amanda Graziela G. Mendes, Carmem D. L. Campos, José L. Pereira-Filho, Viviane S. S. Almeida, Israel V. Moreira, Raphael F. Marques, Mayara Cristina P. Silva, Valério Monteiro-Neto

**Affiliations:** 1 Centro de Ciências Biológicas e da Saúde, Programa de Pós Graduação em Ciências da Saúde, Universidade Federal do Maranhão—UFMA, São Luís 65080-805, MA, Brazil; amanda.graziela@discente.ufma.br (A.G.G.M.); carmem.campos@discente.ufma.br (C.D.L.C.); jlp.filho@discente.ufma.br (J.L.P.-F.); viviane.almeida@discente.ufma.br (V.S.S.A.); israel.moreira@ufma.br (I.V.M.); 2 Centro de Ciências Biológicas e da Saúde, Programa de Doutorado em Biotecnologia—Rede Nordeste de Biotecnologia, Universidade Federal do Maranhão—UFMA, São Luís 65080-805, MA, Brazil; raphael.marques@discente.ufma.br; 3 Centro de Ciências Biológicas e da Saúde, Programa de Pós Graduação em Saúde do Adulto, Universidade Federal do Maranhão—UFMA, São Luís 65080-805, MA, Brazil; mayara.silva@ufma.br

**Keywords:** ellagic acid, antifungal activity, *Candida auris*, synergism, drug delivery

## Abstract

Ellagic acid (EA), a naturally occurring phenolic compound, has garnered significant interest as a potential antifungal agent owing to increasing fungal resistance and a scarce therapeutic pipeline. This review consolidates the evidence of the broad-spectrum activity of EA against critical priority pathogens, including *Candida auris* and *Cryptococcus neoformans*. We highlight its multi-target mechanisms of action, such as the impairment of cell wall integrity and plasma membrane disruption resulting from the inhibition of ergosterol biosynthesis, and inhibition of key enzymes, such as laccase. In addition to its direct growth-inhibitory effects, EA exhibits antivirulence properties, reducing biofilm formation and hyphal morphogenesis. Notably, it demonstrates synergistic potential with conventional antifungals, such as fluconazole, enhancing efficacy and potentially hindering the emergence of resistance. Although its poor solubility and bioavailability pose therapeutic challenges, advanced formulations such as liposomal systems show promise for improving its delivery. We conclude that EA is a promising candidate for developing new antifungal strategies, particularly as a synergistic agent or in nanoformulations, warranting further investigation to translate its potential into clinical practice.

## 1. Introduction

The treatment of fungal infections represents a growing challenge to global public health, in addition to multiple factors, such as the difficulty in diagnosis, high rates of morbidity and mortality, and the ability of these microorganisms to acquire resistance to antifungal drugs [1,2]. Recent estimates indicate 6.5 million annual cases of invasive fungal infections, resulting in 3.8 million deaths. Of these deaths, approximately 2.5 million (68%; range, 35–90%) are directly attributable to fungal infections [3].

In this context, in 2022, the World Health Organization (WHO) published a document with the List of Priority Fungal Pathogens, highlighting fungal species of great concern, including *Cryptococcus neoformans*, *Aspergillus fumigatus*, *Candida auris*, and *Candida albicans*, which were classified as critical priorities. In addition to these, other species were included in the high and medium priority categories, reflecting the diversity and complexity of invasive fungal infections on a global scale [2]. This scenario is aggravated by the scarcity of new antifungal drugs, which significantly limits the available therapeutic options [4,5].

Currently, antifungal therapy faces significant challenges owing to its limited efficacy, adverse effects, and increasing resistance. The five main antifungal classes include allylamines, azoles, echinocandins, polyenes, and pyrimidine analogs, which are limited by the cellular similarity between fungi and humans and the rise in drug resistance [5]. Importantly, these classes possess chemical structures distinct from those of phenolic compounds, such as ellagic acid (EA), underscoring the need for novel therapeutic scaffolds.

According to a recent WHO report, only two antifungal agents remain in phase III of clinical development, highlighting the scarcity of innovative antifungal pipelines [4]. These two candidates are Ibrexafungerp, a triterpenoid indicated for vulvovaginal candidiasis and invasive fungal infections, and Rezafungin, intended for the management of invasive candidiasis and candidemia, as well as for prophylaxis in adults undergoing allogeneic bone marrow transplantation. In addition, much of the research is concentrated in a few countries, such as the United States, Canada, the United Kingdom, Switzerland, China, and Japan, highlighting the need to expand global investments in therapeutic strategies that meet emerging clinical demands and promote greater equity in access to treatment [4].

At the same time, the combination of drugs has been consolidated as a promising alternative in the context of conventional therapies and in the use of natural products with antimicrobial activity. The association of two drugs can result in synergistic or additive interactions, allowing the reduction in the individual concentrations necessary to achieve the desired therapeutic effects. In addition, the use of substances with different mechanisms of action makes it possible to act on several targets, which hinders the development of microbial resistance [6,7].

Therefore, there is a need to explore new drugs and alternative therapies for treating infections [6,8]. In this context, natural products are a traditional and continuous source of bioactive compounds. Characterized by a wide range of biological effects, these compounds serve as relevant molecular prototypes. Despite their potential for toxicity or adverse effects, they remain highly promising for tackling resistant strains and inspiring the development of new drugs, which may possess safety profiles superior to those of available synthetic medications [8].

Ellagic acid (EA) is among the plethora of natural compounds with antimicrobial activities. It is a dilactone of hexahydroxyphenic acid (HHDP), which can be considered a dimeric derivative of gallic acid. EA can be produced in various plants, primarily through the hydrolysis of ellagitannins, a widely distributed group of secondary metabolites [9,10]. EA has been reported to have extensive pharmacological and biological properties, including antibacterial [11], antifungal [12], antioxidant [13], anti-inflammatory [14], antihyperlipidemic [15], antiviral [16], antiangiogenic [17], anti-cancer [18], cardioprotective [19], chemopreventive [20], neuroprotective [21], and antidiabetic [22] properties.

Regarding its antifungal activity, EA has garnered significant attention because of its demonstrated efficacy against a broad spectrum of clinically relevant fungi, including WHO critical priority pathogens such as *Candida auris* and *Cryptococcus neoformans*, coupled with a multi-target mechanism of action that extends beyond that of many other common plant-derived phenolics. This combination of broad-spectrum activity and a potentially resistance-hindering multi-target profile positions EA as a particularly promising candidate for further investigation and development [23,24,25].

Therefore, the primary objective of this review is to critically consolidate and analyze the existing scientific evidence on the antifungal activity of EA, with a specific focus on its multi-target mechanisms of action, its synergistic potential with conventional antifungals, and the advanced formulation strategies developed to overcome its bioavailability limitations. We aim to provide a comprehensive and updated overview that not only summarizes the state-of-the-art but also identifies knowledge gaps and outlines future perspectives for the development of EA as a viable antifungal agent, either as a standalone or adjuvant therapy, in the fight against resistant fungal infections.

For this review, a comprehensive search was conducted in several scientific databases, including PubMed, SciELO, and LILACS, using the Boolean operator “AND”, with the aim of identifying original studies addressing the isolated antifungal activities of EA and/or its formulations. The literature review did not establish restrictions regarding the publication period, allowing for broad temporal coverage. To identify relevant publications, specific search terms were used, such as “ellagic acid AND antifungal activity,” “ellagic acid AND antifungal mechanisms of action,” “antifungal AND interaction with ellagic acid,” and “antifungal resistance AND ellagic acid”.

After the search phase, the studies were carefully analyzed to ensure the quality of the selection. Then, publications that addressed properties of EA unrelated to its antifungal activity against clinical or phytopathogenic pathogens were discarded. Studies that only confirmed the presence of EA in extracts or formulations with antifungal activity, but did not evaluate the compound in isolation, making it impossible to directly attribute the observed effect to EA, were also excluded.

## 2. Chemical Characteristics and Sources of Ellagic Acid

### 2.1. Chemistry of Ellagic Acid

**Figure 1:**
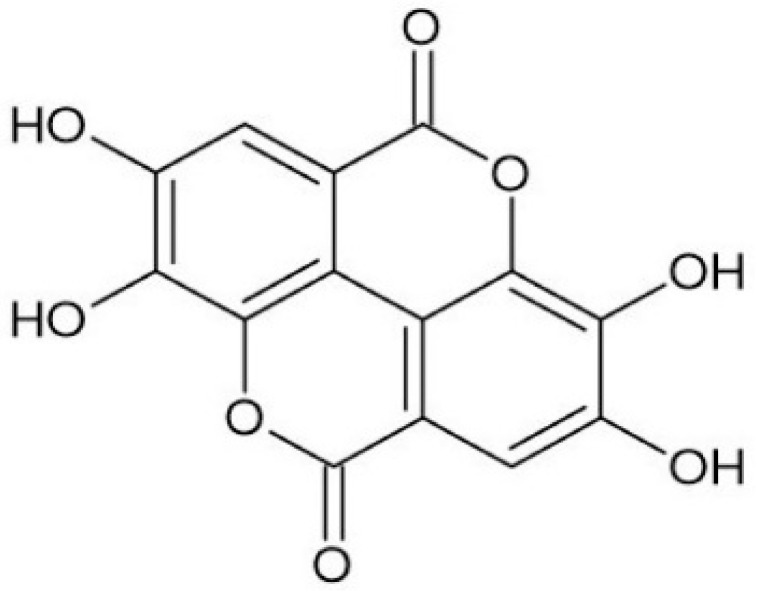
Representation of the chemical structure of ellagic acid. Source: [26].

Ellagic acid (EA; IUPAC name: 2,3,7,8-tetrahydroxy-chromeno[5,4,3-cde]chromene-5,10-dione; molecular formula: C_14_H_6_O_8_; molecular weight: 302.194 g/mol) is a natural phenolic compound classified within the hydrolysable tannin group, specifically as a dilactone derived from hexahydroxydiphenic acid (HHDP) [9,10]. Its chemical structure consists of a rigid, planar system of four fused aromatic rings, which confers significant lipophilicity, along with four phenolic hydroxyl groups and two lactone (ester) moieties that contribute to its hydrophilic character and ability to act as both hydrogen bond donors and acceptors (Figure 1).

This conjugated polyphenolic architecture grants EA considerable intrinsic oxidative stability, a property of dual significance for its pharmaceutical development [26,27]. First, it necessitates careful formulation and storage strategies to prevent potential degradation and ensure the integrity of the dosage form. Second, this stability underlies EA’s potent antioxidant capacity of EA, enabling it to effectively scavenge free radicals and mitigate oxidative stress [13], a beneficial pharmacological effect in the context of infection-associated inflammatory damage.

From a biopharmaceutical perspective, EA is classified as a Class IV compound according to the Biopharmaceutical Classification System (BCS), characterized by low aqueous solubility and low intestinal permeability [28]. These inherent properties, combined with extensive first-pass metabolism, potential hydrolysis and conversion to urolithins in the gut, and rapid systemic elimination, severely limit its oral bioavailability [29] and represent a major hurdle for the translation of its promising in vitro bioactivity into in vivo therapeutic efficacy.

Consequently, a major focus of contemporary research is the development of advanced formulation strategies, such as particle size reduction, solid dispersions, cyclodextrin complexes, and lipid-based or polymeric nanocarriers, to overcome these pharmacokinetic barriers and unlock EA’s full clinical potential of EA [30].

### 2.2. Natural Sources of Ellagic Acid

EA is found in several plants, such as arctic mulberry [31], blackberry [32], pomegranate [33], red raspberry, pecan [32], oak [34], sweet chestnut [35], among others. Table 1 presents a comprehensive list of plant species in which EA has been identified and studied for its antifungal activity. The species are diverse, ranging from well-known sources such as pomegranate (*Punica granatum* L., bark) and chestnut (*Castanea sativa* Miller., bark) to less common plants like *Klainedoxa gabonensis* (stem bark) and *Terminalia albida* (roots). The plant parts utilized vary widely, including the bark, leaves, flowers, fruits, seeds, and aerial parts, highlighting the broad distribution of EA within plant tissues.

In addition to these natural reservoirs, EA can be obtained through various chemical, biomimetic, and enzymatic synthesis routes. These methods have been developed to provide controlled and scalable production of EA and its derivatives. The most common approach is based on the oxidative dimerization of gallic acid or its esters (e.g., methyl gallate), followed by intramolecular lactonization. This process employs various oxidants, such as o-chloranil, cerium ammonium nitrate, ferric chloride, and hypervalent iodine reagents (PIDA and PIFA); however, it often results in moderate yields (10–50%) and requires complex purification steps owing to byproduct formation [61]. Historically, the synthesis of EA dates back to the 19th century, using Löwe’s method with arsenic acid or silver oxide. Recent advances have aimed to improve the efficiency and selectivity of these processes. These include Ullmann-type biaryl couplings and biomimetic strategies that mimic plant biosynthesis to achieve higher yields with fewer steps. Additionally, enzymatic approaches, such as glucansucrase-mediated transglycosylation, enable the synthesis of EA derivatives with enhanced solubility and bioactivity, broadening their potential pharmaceutical and nutraceutical applications [62].

## 3. Antifungal Activity

### 3.1. In Vitro Activity

EA exhibits a considerable spectrum of in vitro antifungal activity, positioning it as a promising natural antifungal agent [23,63,64]. Studies have demonstrated its efficacy against a diverse range of clinical and phytopathogenic strains, as systematically summarized in Table 2. The use of the isolated compound in these assays, as opposed to crude extracts, allows for a more accurate attribution of the observed antifungal effects directly to EA.

Table 2 compiles data on the in vitro antifungal activity of isolated EA, presenting minimum inhibitory concentration (MIC) values (or inhibition zone diameter, IZ) and the methodologies employed. The data reveal that EA is active against several fungal species classified by the WHO as critical or high priority, underscoring its clinical relevance [2].

A critical analysis of the compiled MIC values revealed variability against the same species, particularly for *C. albicans*, where the values ranged from 0.125 to >1000 µg/mL. This disparity is a common challenge in aggregating data from independent studies and can be attributed to several factors [70,71,72], including the following:

i. Differences in the source and purity of EA;
ii. Variations in susceptibility testing methodologies (e.g., broth microdilution vs. agar diffusion);
iii. Differences in growth medium, incubation time, and inoculum size;
iv. Inherent variability in membrane composition and efflux pump activity among different clinical isolates.

Among clinically relevant fungi, *Candida* is the most extensively studied. Activity has been reported against *C. albicans* [12,23,24,43,45,49,56,66,68,69] and numerous non-*albicans Candida* species (NAC), including the critical priority pathogen *C. auris* [24,45], as well as *C. glabrata* [24,45,46,66,67], *C. krusei* [24,43,46,49,67], *C. tropicalis* [24,49,66,67], *C. parapsilosis* [24,43,46,49,67,69], *C. zeylanoides* [69], *C. dubliniensis* [67], and *C. kefyr* [46].

In addition to *Candida* species, EA has also shown activity against yeasts such as *C. neoformans* [43,49,63,65,69] and *S. cerevisiae* [42,45]; dermatophytes including *Trichophyton rubrum*, *Trichophyton mentagrophytes*, *Trichophyton violaceum*, *Trichophyton schoenleinii*, *Trichophyton verrucosum*, and *M. canis* [66]; and non-dermatophyte filamentous fungi such as *Aspergillus brasiliensis*, *Aspergillus candidus* [69], *Aspergillus flavus*, *Aspergillus ochraceus*, *Aspergillus niger*, *Penicillium ochrochloron*, *Penicillium funiculosum* [56], and *Mucor miehei* [48].

Furthermore, EA is active against important phytopathogenic fungi that pose a significant threat to agriculture. Controlling these pathogens is essential to minimizing productivity loss and ensuring food security. Sensitive species included *Alternaria alternata*, *Botrytis cinerea*, *Colletotrichum acutatum*, and *Coniella granati* [69]. The emergence of triazole resistance in agroecosystems, with mechanisms potentially cross-reacting with clinical antifungals, highlights the importance of exploring alternative compounds such as EA [73].

Beyond standardized commercial preparations, EA has been purified directly from specific plant sources in laboratory settings, confirming its role as a widely distributed and isolable antifungal agent. The studies included in Table 2 demonstrate that EA isolated from different plant parts, such as the leaves of *Excoecaria lucida* [46] and *Eugenia uniflora* [45], the bark of *Lafoensia pacari* [49], and the stem bark of *Klainedoxa gabonensis* [48], retains antifungal activity against clinically relevant pathogens, including non-*albicans Candida* (NAC) species. This consistent bioactivity, whether from commercial vendors or direct botanical isolation, underscores the intrinsic nature of EA’s antifungal properties, independent of its immediate purification pathway.

However, it is crucial to distinguish studies evaluating purified compounds from those on crude plant extracts. In crude extracts, EA is present within a complex phytochemical matrix containing flavonoids, tannins, and other phenolic compounds. In such mixtures, the observed bioactivity is a composite effect, where EA may act additively or synergistically with other constituents, potentially leading to an efficacy profile distinct from that of the isolated compound [38,40,51,58,60]. Therefore, while the successful laboratory isolation of active EA from various plants validates its ecological role as a phytoalexin and confirms it as a key antifungal principle, studies on crude extracts represent a complementary approach. This approach aims to understand the whole-plant therapeutic potential or discover new synergistic combinations within the natural matrix. Future studies employing bioassay-guided fractionation of highly active crude extracts could further elucidate the specific contributions of EA and help identify other complementary antifungal compounds.

In addition to the standard MIC determination and agar diffusion assays detailed in Table 2, more dynamic assays were implemented to further investigate the antifungal properties of EA. Growth and killing kinetics studies have provided deeper insights into their modes of action. A growth curve experiment revealed that EA at 4 µg/mL induced maximum inhibition of *C. albicans* growth at 14 h, whereas concentrations below 2 µg/mL had no effect [74]. This fungistatic nature was corroborated by elimination kinetics assays, which demonstrated that EA exerts a primarily fungistatic effect against both *C. albicans* and the critical pathogen *C. auris* [24], a profile it shares with azole antifungals, such as fluconazole, at their typical fungistatic concentrations. However, it is important to note that fluconazole can exhibit dose-dependent fungicidal activity at substantially higher concentrations [75]. For *Cryptococcus neoformans*, time-kill studies have consistently shown that EA’s activity of EA is both time- and dose-dependent [63,65], confirming a dynamic interaction in which efficacy increases with concentration and exposure time.

Further investigations into time-dependent susceptibility have yielded nuanced results. When evaluating pomegranate-derived compounds, EA exhibited superior activity against the reference strain *C. albicans* SC5314 than a clinical isolate after a prolonged 72 h exposure [69]. In a host–pathogen interaction model, the ability of EA to reduce the invasion of Caco-2 epithelial cell layers by *C. albicans* was tested. Although a trend towards reduced invasion was observed, the difference compared to the untreated control did not reach statistical significance (*p* = 0.4289) [23].

The effects of EA on fungal morphology and viability have been vividly demonstrated in studies using the dermatophyte *Trichophyton rubrum*. Scanning electron microscopy revealed that EA treatment caused significant alterations (*p* < 0.05) in hyphal size, shape, and overall biomass [76]. Furthermore, EA induced apoptosis-like programmed cell death in *T. rubrum*; treatment for over 7 days led to a dose-dependent decrease in normal cells and a concomitant increase in apoptotic cells. This effect directly correlates with a loss of viability, as evidenced by inhibition rates of 65.34%, 67.40%, and 72.88% at concentrations of 64, 128, and 256 μg/mL, respectively, confirming its potent fungicidal activity against this dermatophyte [76].

### 3.2. In Vivo Activity

The in vivo efficacy of EA has been validated across a spectrum of fungal infection models and animal hosts, as shown in Table 3. These studies demonstrate the consistent protective effect and therapeutic potential of EA.

In a dermatophytosis model, topical application of EA (4.0 and 8.0 mg/cm^2^ for 14 days) promoted complete clinical and mycological cure of *Trichophyton rubrum* infection in guinea pigs, showing efficacy comparable to the standard drug terbinafine [66]. Against systemic infections caused by critical priority pathogens, EA significantly improved survival in invertebrate models. In *Galleria mellonella* larvae infected with *C. auris*, a single pre-treatment dose of EA (32 mg/kg) increased survival by 44% and prolonged the survival time (*p* = 0.0023) [24]. Similarly, EA administration enhanced the survival of *Caenorhabditis elegans* infected with *C. albicans* [24] and *Drosophila melanogaster* against a *C. albicans* challenge, without evidence of host toxicity at the effective dose [25].

Most notably, in a clinically relevant model of systemic cryptococcosis in immunocompromised (leukopenic) mice, EA treatment at 40 mg/kg conferred a substantial survival benefit (30% vs. 0% in saline-treated controls at 40 days post-infection) and reduced the lung fungal burden [63].

Collectively, these findings from diverse experimental systems support the in vivo antifungal efficacy of EA, highlighting its potential against superficial mycoses and severe systemic fungal infections.

### 3.3. Antivirulence Activity

In addition to its direct growth-inhibitory effects, EA disrupts key fungal virulence factors, compromising pathogenicity and persistence. These factors include morphological transition, biofilm formation, and secretion of tissue-damaging enzymes [77,78].

i. Morphological Transition and Hyphal Inhibition: The yeast-to-hypha transition is critical for *C. albicans* adhesion, invasion, and tissue penetration [79,80]. While EA alone showed only a modest effect on hyphal extension [24], its combination with fluconazole induced a significant reduction in hypha formation compared to the untreated control (*p* < 0.05), indicating that EA can potentiate the inhibition of this key morphogenetic process under specific conditions [12].
ii. Biofilm Disruption: Biofilm formation is a major therapeutic challenge, as it confers protection against antifungals and host defenses [80,81]. EA exhibits promising antibiofilm properties. It prevents biofilm formation in *C. auris* and *C. albicans* [24] and, remarkably, eradicates pre-formed biofilms of *C. neoformans* by up to 91% [63]. Against *C. albicans* biofilms, EA, alone or in combination with fluconazole, shows efficacy against early-stage structures; however, the eradication of mature biofilms requires much higher concentrations, reaching up to eight times the MIC [12]. However, this stage-dependent activity presents a challenge when comparing different experimental models. While one study found that higher EA concentrations were needed to impact more established biofilms [12], another reported that EA significantly reduced viability (*p* < 0.05) in 48 h-old biofilms but not in 24 h-old ones [68]. This apparent discrepancy likely stems from differences in biofilm maturation models, definitions of “mature” biofilms, and the specific metabolic or architectural characteristics of biofilms at these precise time points, highlighting the complex and context-dependent nature of EA’s antibiofilm activity.
iii. Inhibition of Hydrolytic Enzymes: EA also targets enzymatic virulence factors. Although it does not inhibit *Candida* proteinase production, EA significantly reduces phospholipase secretion (*p* < 0.05) in *C. auris*, a critical factor for host cell damage and invasion [24].

These findings demonstrate that EA possesses a broad-spectrum antivirulence profile. By interfering with hyphal morphogenesis, biofilm integrity, and phospholipase activity, EA undermines multiple pathways essential for fungal pathogenicity and persistence, highlighting its potential as an agent that could mitigate damage and improve therapeutic outcomes.

### 3.4. Synergistic Interactions

The potential of EA to enhance the efficacy of existing antifungal agents has been explored through combination studies, yielding variable yet promising results. When evaluated against *C. strains* in checkerboard assays, EA showed indifferent interactions (no synergy or antagonism) when combined with amphotericin B, caspofungin, or fluconazole [24]. However, a more strain-specific analysis revealed that EA can potentiate fluconazole. Mendes et al. (2024) reported that the combination of EA and fluconazole resulted in synergistic, additive, and indifferent interactions against 54.5%, 27.3%, and 18.2% of the tested *C. albicans* strains, respectively, suggesting that EA can resensitize a significant proportion of isolates, potentially including resistant phenotypes [12]. This 54.5% synergy rate can be contextualized within the landscape of natural product-azole combinations, which often yield variable synergistic outcomes, as previously reported. For instance, semisynthetic derivatives of eugenol (eugenol-tosylate and its congeners) exhibited a 36% synergy rate (FICI ≤ 0.5) when combined with fluconazole against a panel of clinical *C. albicans* isolates [82]. In another study, the triterpenoid asiatic acid demonstrated a potent synergistic effect (FICI = 0.25) with fluconazole against *C. albicans* strains (50% synergy rate), reducing the fluconazole MIC from >512 to 0.25–0.5 µg/mL [83]. Although direct quantitative comparisons are nuanced because of differences in the strain panels and experimental designs, these examples illustrate a spectrum of synergistic potential. The 54.5% synergy rate reported for EA positions it as a highly promising candidate among natural compound potentiators, especially considering its capacity to resensitize the majority of tested isolates, a characteristic crucial for overcoming antifungal resistance.

Beyond conventional antifungals, EA demonstrates a clear synergy with other bioactive phenolic compounds. A pronounced synergistic effect (FICI = 0.25) was observed between EA and caffeic acid phenethyl ester (CAPE) against *C. albicans*, drastically reducing the MIC of both compounds and inhibiting microbial growth within 14 h [48]. Synergy has also been reported with tacrolimus in a fission yeast model [84]. Conversely, combinations of EA with the plant metabolites myricitrin or gallic acid did not demonstrate formal synergy (based on FICI analysis) but showed varying MIC values against *C. albicans*, *C. glabrata*, and *C. auris* [45].

The divergent outcomes of these combination studies, ranging from strong synergy to indifference, likely stem from both biological and methodological factors. The synergy between EA and fluconazole appears to be strain-dependent, potentially linked to the ability of EA to target efflux pumps or compromise membrane integrity in resistant isolates [12]. Methodologically, the interpretation of synergy is sensitive to the criteria used for the Fractional Inhibitory Concentration Index (FICI) and can be influenced by technical challenges, such as the poor aqueous solubility of EA, which may limit its observed bioavailability in in vitro assays [23,77]. These variable results underscore a significant opportunity, highlighting the need for systematic, well-controlled investigations to define the specific strains, compound ratios, and experimental conditions that reliably unlock the synergistic potential of EA-based combinations, paving the way for more effective therapeutic strategies.

## 4. Mechanisms of Action

**Figure 2:**
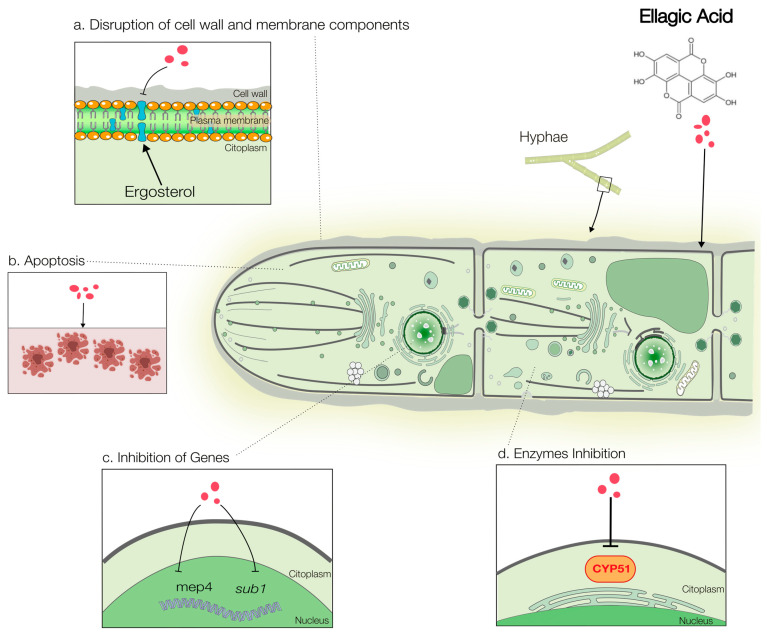
Schematic representation of the potential antifungal mechanisms of EA against *Trichophyton rubrum*. EA acts on *T. rubrum* through a coordinated mechanism involving: (**a**) impairment of membrane integrity by inhibiting ergosterol biosynthesis, interrupting the production of this essential sterol of the fungal cell membrane; (**b**) induction of programmed cell death (apoptosis) in fungi as a therapeutic strategy, reducing the persistence of infection, since it forces the fungus to self-destruct; (**c**) modulation of the expression of genes associated with virulence (*mep4*, *sub1*), decreasing the fungus’s ability to invade, survive and cause disease; (**d**) inhibition of enzymes (CYP51), which is one of the main antifungal therapeutic targets, as it catalyzes the conversion of lanosterol to ergosterol, which is the main sterol of the fungal membrane. Created using Servier Medical Art (https://smart.servier.com/), licensed under CC BY 4.0.

**Figure 3:**
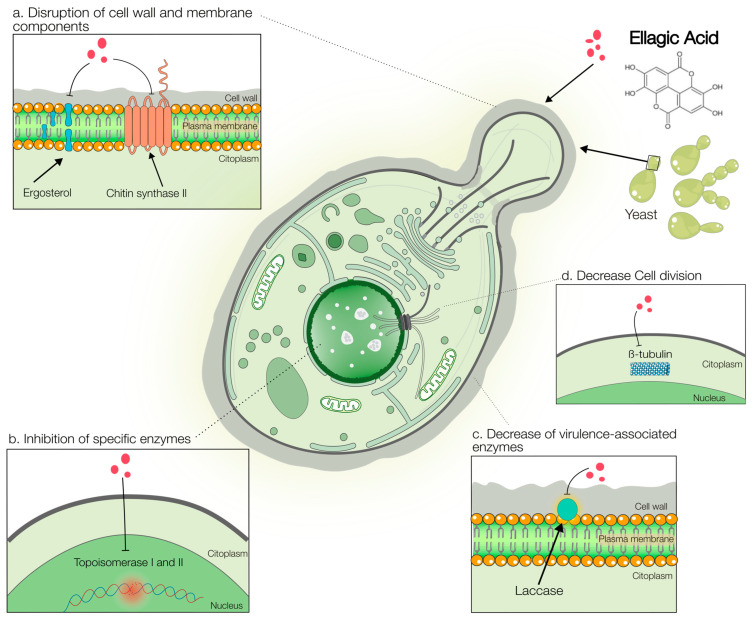
Schematic representation of the proposed antifungal mechanisms of EA against pathogenic yeasts. EA exerts its antifungal effects through several targeted actions: (**a**) disruption of essential cell structures by targeting the cell wall (e.g., Chitin Synthase II) and cell membrane (e.g., ergosterol biosynthesis). (**b**) Inhibition of key enzymes, such as topoisomerase I/II, which interfere with DNA replication and repair. (**c**) Attenuation of virulence by inhibiting laccase, a critical enzyme for melanin production in *C. neoformans*. (**d**) Potential disruption of cell division via predicted binding to β-tubulin, a key cytoskeletal protein. Created using Servier Medical Art (https://smart.servier.com/), licensed under CC BY 4.0.

The antifungal activity of EA stems from a multi-target mechanism that disrupts critical cellular processes in pathogenic fungi. The evidence supporting these mechanisms varies in robustness, ranging from direct biochemical validation to preliminary in silico predictions. This section consolidates these mechanisms, distinguishing between those well-characterized in filamentous fungi, such as *Trichophyton rubrum* (Figure 2), and those primarily elucidated in yeasts (Figure 3).

### Well-Validated Mechanisms: Cell Membrane and Cell Wall Disruption

One of the most robustly supported mechanisms is the disruption of ergosterol biosynthesis. In *T. rubrum*, direct biochemical evidence shows that EA significantly reduces cellular ergosterol content and inhibits CYP51 (14-α-demethylase), a key enzyme in the ergosterol pathway [66,76]. This disruption correlated with the induction of apoptosis and downregulation of virulence-associated genes (*mep4* and *sub1*), providing compelling genetic support for this mechanism (Figure 2).

Similarly, strong functional evidence indicates that EA compromises the integrity of the cell wall of yeast cells (Figure 3a). This was demonstrated by the sorbitol protection assay, where the MIC of EA increased significantly in osmotically stabilized media for both *Candida* spp. (from 0.125–0.5 to 64 µg/mL) [24] and *S. cerevisiae* (from 62 to 250 µg/mL) [49]. For instance, the MIC against *C. auris* increased 128-fold in the presence of sorbitol (64 µg/mL) [24]. This functional evidence is further strengthened by the fact that EA activity was not affected in ergosterol binding assays, clearly distinguishing its mechanism from that of polyenes, such as amphotericin B [24,49].

For other targets, such as Chitin Synthase II, the evidence is more preliminary or suggests a secondary role. Direct biochemical assays have confirmed that EA can inhibit specific enzymes, although the physiological relevance may vary. For instance, EA demonstrated inhibitory activity against Chitin Synthase II from *S. cerevisiae*, with a reported IC_50_ of 149 µM [79]. While this constitutes direct in vitro evidence, the relatively high concentration required and the need for studies on pathogenic fungi mean that its functional importance in EA’s overall antifungal effect warrants further investigation (Figure 3a).

A similar case was observed for DNA topoisomerases I and II. Brighenti et al. reported that EA inhibited these enzymes with IC50 values of 56.6 and 53.9 µg/mL, respectively [69]. However, these values are relatively high compared to the MICs of EA against many fungi. This discrepancy suggests that while topoisomerase inhibition occurs in vitro, it is likely a secondary mechanism contributing to the antifungal effect rather than the primary cause of cell death at clinically relevant concentrations (Figure 3b).

The best-validated specific enzyme target for EA is laccase in *C. neoformans*. This enzyme is critical for melanin synthesis, a key virulence factor that protects the pathogen from oxidative stress and immune defenses, facilitating its survival and replication within host cells, including in the brain [85,86,87]. Azam et al. (2022) [65] provided multifaceted evidence for EA’s action on this target, progressing from in silico molecular docking to in vitro enzymatic inhibition. Crucially, they demonstrated the functional consequences of this inhibition, including reduced melanization and impaired proliferation within macrophages [65]. These findings, which directly link laccase inhibition to a loss of virulence, reveal the molecular basis of the interaction between EA and laccase and underscore its potential as an anti-cryptococcal agent (Figure 3c).

In contrast, the proposed interaction with β-tubulin remains a computationally derived hypothesis. In silico docking studies suggest that EA can replicate the critical interactions of griseofulvin with β-tubulin, indicating its potential to disrupt fungal cytoskeleton formation [88]. While this prediction is valuable for generating new research avenues, it represents the most preliminary level of evidence among proposed mechanisms. Experimental validation through in vitro tubulin polymerization assays or genetic studies is essential to confirm this target (Figure 3d).

In summary, the robustness of the evidence for antifungal mechanisms is decidedly target-dependent. The disruption of the cell membrane via ergosterol biosynthesis and compromise of cell wall integrity are supported by strong functional and biochemical data. Among the specific enzyme targets, laccase inhibition is particularly well validated. In contrast, the inhibition of chitin synthase and topoisomerases, although experimentally demonstrated, may play a secondary role, and the proposed interaction with β-tubulin awaits experimental confirmation. This multi-target profile, encompassing both well-validated and potential mechanisms, underpins EA’s promise of EA as an antifungal agent and guides future research priorities.

## 5. Cytotoxicity and Safety Profile

A favorable safety profile is a critical attribute of any promising therapeutic candidate. Preclinical evaluations of EA across diverse models indicate its low intrinsic cytotoxicity and potential protective effects against drug-induced organ damage.

In vitro cytotoxicity assessments have consistently reported low toxicity of EA. Mammalian erythrocytes serve as a sensitive model for assessing membrane damage [89,90]. Hemolysis assays using this model confirmed that EA does not damage human erythrocyte membranes across a wide concentration range (0.125–8000 µg/mL) [12]. Similarly, EA showed no cytotoxicity against human lung fibroblasts (MRC-5-SV2) or murine macrophages (RAW 264.7), even at high concentrations (up to 2048 µg/mL) [46]. In other adherent cell lines, EA exhibited minimal-to-moderate cytotoxicity in a dose- and time-dependent manner. In HeLa cells, most treatments were classified as low cytotoxic [12], whereas in 3T3 fibroblasts, moderate cytotoxicity (59% viability) was observed only at a high concentration of 250 µg/mL [68].

The in vivo tolerance and protective effects further support the safety of EA. In invertebrate models, EA administration (up to 128 mg/kg) caused no acute toxicity in *G. mellonella* larvae [24] and was non-toxic at therapeutic doses in *D. melanogaster* [25]. Notably, studies in mammalian models have highlighted its beneficial protective role. EA co-administration mitigated cyclosporine A-induced oxidative damage and tissue injury in the liver, heart, and kidney of rats [91]. In a model of systemic cryptococcosis with immunosuppression, both EA and its liposomal formulation (Lip-EA) alleviated cyclophosphamide-induced renal and hepatic toxicity, normalizing key biomarkers such as AST, ALT, BUN, and serum creatinine [63].

In summary, the collective evidence positions the EA favorably. It demonstrates low direct cytotoxicity in vitro, excellent tolerance in vivo, and protective properties against toxicity caused by other drugs. This preliminary safety profile, especially when contrasted with the renal toxicity associated with current azoles and polyenes [63], underscores the potential of EA as a promising and potentially safer antifungal candidate, warranting further investigation.

## 6. Therapeutic Challenges and Technological Advances

### 6.1. The Bioavailability and Solubility Challenge

EA has significant ethnopharmacological relevance and is traditionally used in preparations such as pomegranate tea for wound healing and inflammation. However, its therapeutic translation faces a major pharmacokinetic hurdle: extremely low aqueous solubility (≈9.7 µg/mL at 37 °C) and consequent poor oral bioavailability, resulting in minimal systemic plasma concentrations (≈120 ng/mL) [29,92]. This poor biopharmaceutical profile limits dissolution, leads to variable absorption, and restricts the attainment of therapeutic tissue levels, despite promising in vitro activity [29].

**Figure 4:**
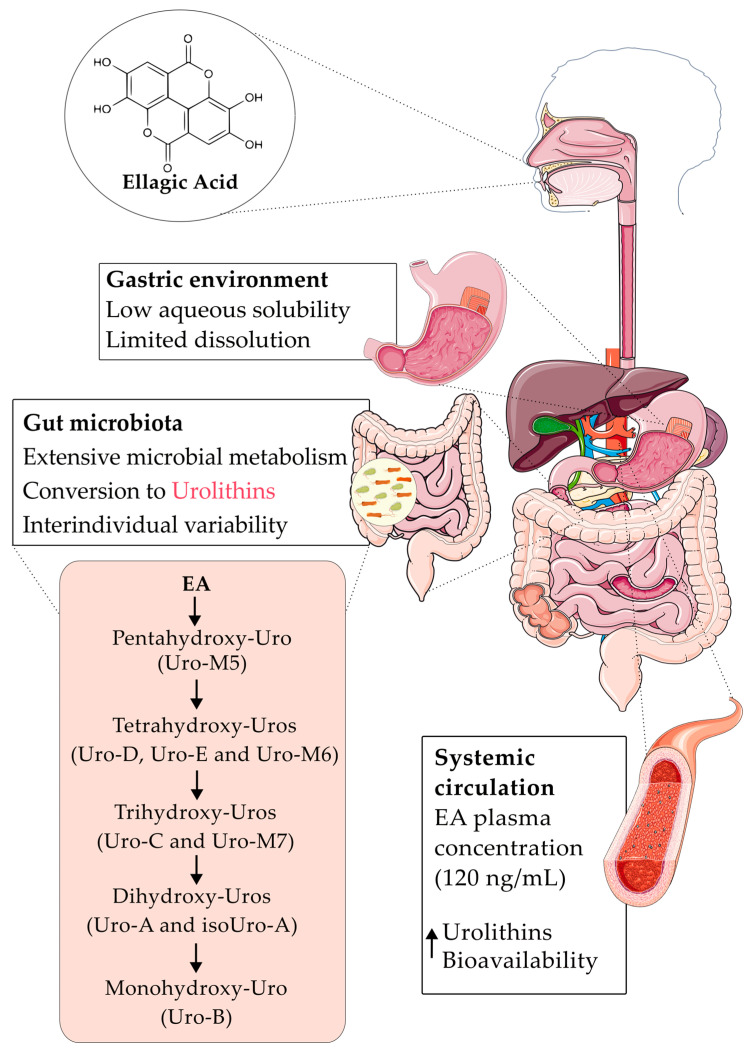
Gut microbiota-mediated metabolism of ellagic acid into bioavailable urolithins. Following oral ingestion, the poor solubility of native EA limits its dissolution and absorption in the gastrointestinal tract. In the intestine, particularly the colon, microbial enzymes convert EA into a series of urolithin metabolites (e.g., Uro-A and Uro-B) via dehydroxylation and lactone cleavage. These urolithins possess significantly higher bioavailability (indicated by ↑) and are responsible for the systemic biological activities attributed to EA. Created using Servier Medical Art (https://smart.servier.com/), licensed under CC BY 4.0.

Notably, the biological activity of EA in vivo is largely mediated by its metabolism in the body. Upon ingestion, EA is extensively biotransformed by specific families of the gut microbiota (e.g., Eggerthellaceae, Lactobacillaceae) into a series of bioavailable metabolites known as urolithins (e.g., Urolithin A, B) [93,94]. This crucial metabolic pathway, which overcomes the limitations of the parent compound, is illustrated in Figure 4.

The microbial transformation of EA involves sequential lactone ring cleavage, decarboxylation, and dehydroxylation, yielding various urolithins with different hydroxylation patterns (e.g., from pentahydroxy-Uro-M5 to monohydroxy-Uro-B) [95]. This conversion occurs predominantly in the distal colon and varies between individuals, defining distinct urolithin metabotypes [96]. Crucially, these urolithin metabolites exhibit 25–80 times greater systemic bioavailability and absorption than EA itself, and they possess unique biological activities, effectively explaining the systemic effects observed after EA consumption [97].

Therefore, the key challenge is not merely identifying an effective dose in vitro but developing advanced delivery strategies capable of overcoming these intrinsic pharmacokinetic barriers. Enhancing the solubility and bioavailability of EA through formulation science is essential to unlock its full therapeutic potential [30].

### 6.2. Promising Formulation Strategies

To overcome the pronounced bioavailability limitations of EA, advanced delivery systems have been engineered. These strategies aim to enhance solubility, enable targeted delivery, and ultimately translate in vitro efficacy into therapeutic outcomes in vivo. This is particularly critical for treating opportunistic fungal infections in immunocompromised patients, a population with limited treatment options and high vulnerability [98,99].

i. Nanocarrier Systems for Systemic Delivery: Encapsulation of EA into nanocarriers represents a powerful approach for systemic therapy. The most compelling evidence comes from a liposomal formulation (Lip-EA). In a murine model of systemic cryptococcosis in immunocompromised hosts, intraperitoneal administration of Lip-EA (40 mg/kg) achieved a 70% survival rate and significantly reduced the lung fungal burden (*p* < 0.001), outperforming fluconazole (20% survival) [63]. This demonstrates the potential of nanocarriers to rescue the in vivo efficacy of EA against severe disseminated infections. Another nanotechnological approach, gallium nanoparticles coated with EA (EA-GaNPs), has shown preliminary antifungal activity against *Aspergillus terreus* [100].
ii. Complexation and Solubility Enhancement: Cyclodextrins (CDs) are well-established excipients for improving drug solubility and stability, with a proven track record in commercial antimicrobial formulations [101,102,103]. The complexation of EA with hydroxypropyl-β-cyclodextrin (HP-β-CD) successfully increased its aqueous solubility [23]. Although this did not consistently lower the MIC in vitro [23], the EA/HP-β-CD complex demonstrated significant in vivo activity (*p* < 0.0001) in a murine model of oral candidiasis, reducing hyphal invasion and tissue damage [68]. This highlights that improved solubility can translate to enhanced biological activity in relevant infection models, even without a drastic change in the standard in vitro susceptibility.
iii. Polymeric Systems for Local/Topical Application: For localized infections, polymeric matrices offer controlled release and mucosal adhesion properties. Gellan gum (GG) hydrogels loaded with EA (and its combination with caffeic acid phenethyl ester, CAPE) were effective against *C. albicans* biofilms, inhibiting hyphal formation and showing high biocompatibility. These formulations provided sustained EA release, making them promising candidates for the topical treatment of oral candidiasis [74]. This aligns with the strategic rationale for developing topical EA products, given its low systemic absorption after oral administration [104], a characteristic shared with successful topical antifungals, such as miconazole and nystatin [105,106]. Therefore, owing to its inherent pharmacokinetic profile, EA is a prime candidate for development as a topical antifungal agent [23]. The feasibility of non-oral routes is further supported by studies showing improved bioavailability of EA via subcutaneous delivery [30].
iv. Strategic Outlook: The choice of formulation is critically guided by the target infection. As summarized in Table 4, nanocarriers, such as liposomes, are ideal for severe systemic diseases, whereas biocompatible hydrogels are suited for localized mucosal infections. Cyclodextrin complexes are versatile tools for enhancing solubility across various applications. This portfolio of advanced formulations effectively decouples EA’s potent antifungal pharmacology of EA from its poor pharmacokinetics, paving a concrete path toward its clinical development.

## 7. Conclusions and Future Directions

EA is firmly established as a promising natural antifungal agent. This review consolidates evidence demonstrating its broad-spectrum in vitro activity against significant human pathogens, including WHO critical priority fungi such as *C. auris* and *C. neoformans*, and phytopathogens. In addition to its direct fungistatic and fungicidal effects, EA exhibits compelling antivirulence properties, disrupting key processes such as biofilm formation, hyphal morphogenesis, and melanin production. Its multi-target mechanism of action, which includes disrupting cell wall integrity, inhibiting ergosterol biosynthesis, and targeting specific enzymes (e.g., laccase), presents a significant strategic advantage in overcoming and preventing fungal resistance. Furthermore, EA has demonstrated a favorable preliminary safety profile across various models, showing low hemolytic activity and negligible toxicity in invertebrates, which is crucial for further development.

However, the translation of this promising in vitro activity into a clinically viable therapy is impeded by significant pharmacokinetic challenges, primarily due to its profound aqueous insolubility and consequent poor oral bioavailability. While strategies such as liposomal encapsulation (Lip-EA) and cyclodextrin complexation represent crucial proof-of-concept advances, future research must build upon these findings. Prioritizing the development and systematic comparison of advanced delivery systems, including polymeric nanoparticles, solid dispersions, and self-emulsifying drug delivery systems (SEDDS), is essential for improving solubility, enabling targeted delivery, and providing sustained release profiles.

The observed synergy between EA and fluconazole against a significant proportion of *C. albicans* strains (as discussed in Section 3.2) opens a highly promising therapeutic avenue. The mechanistic basis of this synergy warrants an in-depth investigation to determine whether EA acts as a chemosensitizer by inhibiting efflux pumps, compromising membrane integrity, or disrupting stress response pathways. This understanding should be extended to combinations with other antifungal classes (e.g., echinocandins and polyenes) to explore broad-spectrum synergistic partnerships that could lower therapeutic doses and resensitize resistant isolates.

Crucially, promising data from invertebrate models (*G. mellonella*, *C. elegans*, and *D. melanogaster*) must be validated in mammalian systems. There is a pressing need for comprehensive in vivo efficacy studies using immunocompetent and immunocompromised murine models of invasive candidiasis, aspergillosis, and cryptococcosis. These studies should assess the survival, fungal burden, pharmacokinetics, and biodistribution of new EA formulations. Furthermore, long-term in vivo safety and drug–drug interaction potential must be thoroughly evaluated to satisfy regulatory standards.

In conclusion, although the journey from a natural compound to a registered drug is long, EA possesses a compelling combination of broad-spectrum activity, multitarget action, and a favorable safety profile. By strategically addressing its pharmacokinetic limitations, elucidating synergistic partnerships, and conducting rigorous preclinical validation, EA could transcend its status as a promising natural compound and emerge as a much-needed novel antifungal agent or powerful adjuvant in the global fight against fungal resistance.

## Figures and Tables

**Table 1:** Plant species with identified ellagic acid content and reported antifungal activity.

Scientific Name	Plant Material	References
*Anogeissus latifolia*	Bark	[36]
*Buchenavia tomentosa*	Leaves	[37]
*Carya illinoensis*	Leaves	[38]
*Carya illinoinensis* and *Castanea sativa*	Flowers	[39]
*Caryocar brasiliense*	Bark	[40]
*Castanea sativa* Miller.	Bark	[41]
*Crocus sativus* L.	Stigmata, tepals, and leaves	[42]
*Dimocarpus longan* Lour.	Seed	[43]
*Eucalyptus camaldulensis* L., *Eucalyptus camaldulensis* var. *obtusa* and *Eucalyptus gomphocephala*	Leaves	[44]
*Eugenia uniflora* Linn.	Leaves	[45]
*Excoecaria lucida* Sw.	Leaves	[46]
*Geum aleppicum* Jacq.	Aerial parts (flowers, leaves and stems)	[47]
*Klainedoxa gabonensis*	Stem bark	[48]
*Lafoensia pacari* A. St.-Hil.	Stem bark	[49]
*Lawsonia inermis* L.	Leaves	[50]
*Myrcia hatschbachii*	Stem	[51]
*Nephelium lappaceum* L.	Bark	[52]
*Plinia peruviana *(Poir.) *Govaerts*	Bark	[53]
*Potentilla fruticosa*, *Potentilla glabra* and *Potentilla parvifolia*	Leaves	[54]
*Punica granatum* L.	Bark	[55]
*Quercus robur*, *Quercus macrocarpa* and *Quercus acutissima*	Bark	[56]
*Rhaphiodon echinus* (Nees & Mart.)	Leaves	[57]
*Rhaphiolepis indica* L.	Leaves	[58]
*Terminalia albida*	Roots	[59]
*Zizyphus spina-christi* L.	Leaves and fruits	[60]

**Table 2:** In vitro antifungal activity of ellagic acid against diverse fungal strains.

Origin of EA	Fungus (No. of Strains)	Method *	Activity * (MIC, Unless Noted)	Reference
Commercial source				
Sigma-Aldrich	*Cryptococcus neoformans* (clinical)	MD	16 µg/mL	[65]
	*Candida albicans* SC5314 (ATCC MYA-2876)	MD	0.5 µg/mL	[24]
	*Candida auris* (10 clinical)	MD	0.125–0.25 µg/mL	
	*Candida glabrata* ATCC 90030	MD	0.125 µg/mL	
	*Candida krusei* ATCC 6258	MD	0.125 µg/mL	
	*Candida parapsilosis* ATCC 22019	MD	0.25 µg/mL	
	*Candida tropicalis* ATCC 13803	MD	0.25 µg/mL	
	*C. neoformans* (clinical)	MD	16 µg/mL	[63]
	*C. albicans* ATCC 90028	AD	IZ: 8.75 ± 0.50 mm (1 mg disc)	[43]
	*C. neoformans* (clinical)	AD	IZ: 15.50 ± 1.52 mm (1 mg disc)	
	*C. krusei* ATCC 6258	MD	125 µg/mL	
	*C. parapsilosis* ATCC 20019	MD	7.81 µg/mL	
	*C. albicans ATCC* 90028	MD	62.50 µg/mL	
	*C. albicans* (5 clinical)	MD	7.81–125 µg/mL	
	*C. neoformans* (4 clinical)	MD	15.63–62.50 µg/mL	
	*C. albicans* ATCC 90028	MD	500 µg/mL	[12]
	*C. albicans* SC5314 (ATCC MYA-2876)	MD	250 µg/mL	
	*C. albicans* (21 clinical)	MD	250–2000 µg/mL	
	*Aspergillus flavus* ATCC 9643	MD	0.15 ± 0.01 mg/mL	[56]
	*Aspergillus ochraceus* ATCC 12066	MD	0.22 ± 0.03 mg/mL	
	*Aspergillus niger* ATCC 6275	MD	0.13 ± 0.01 mg/mL	
	*C. albicans* ATCC 12066	MD	0.30 ± 0.03 mg/mL	
	*Penicillium ochrochloron* ATCC 48663	MD	0.12 ± 0.01 mg/mL	
	*Penicillium funiculosum* ATCC 56755	MD	0.23 ± 0.02 mg/mL	
Shanghai Yuanye Biotech	*C. albicans* (2)	MD	25.0–25.0 µg/mL	[66]
	*C. glabrata* (2)	MD	>100.0 µg/mL	
	*C. tropicalis* (2)	MD	50.0–100.0 µg/mL	
	*Microsporum canis* (3)	MD	25.0–100.0 µg/mL	
	*Trichophyton mentagrophytes* (6)	MD	6.25–100.0 µg/mL	
	*Trichophyton rubrum* (6)	MD	6.25–50.0 µg/mL	
	*Trichophyton schoenleinii* (3)	MD	12.5–50.0 µg/mL	
	*Trichophyton verrucosum* (3)	MD	25.0–50.0 µg/mL	
	*Trichophyton violaceum* (6)	MD	25.0–50.0 µg/mL	
Cayman Chemical	*C. albicans* ATCC 18804	MD	1 mg/mL	[67]
	*Candida dubliniensis* NCPF 3108	MD	1 mg/mL	
	*C. glabrata* ATCC 90030	MD	0.004 mg/mL	
	*C. krusei* ATCC 6258	MD	0.125 mg/mL	
	*C. tropicalis* ATCC 13803	MD	1 mg/mL	
	*C. parapsilosis* ATCC 22019	MD	1 mg/mL	
	*C. albicans* SC5314 (ATCC MYA-2876)	MD	50 µg/mL	[23]
	*C. albicans* ATCC 18804	MD	25 µg/mL	[68]
Aktin Chemicals Inc.	*Alternaria alternata* (isolated from infected pomegranates)	MD	165.4 µM	[69]
	*Aspergillus brasiliensis* ATCC 16404	MD and AD	662 µM, IZ: 25 mm (62.5 µg disc)	
	*Aspergillus candidus* (2 clinical)	MD and AD	662 µM, IZ: 21–25 mm (62.5 µg disc)	
	*Botrytis cinérea* (isolated from infected pomegranates)	MD	165.4 µM	
	*C. albicans* ATCC 10321	MD and AD	2.5 µM, IZ: 32 ± 0.1 mm (62.5 µg disc)	
	*C. albicans* SC5314 (ATCC MYA-2876)	MD and AD	2.5 µM, IZ: 34 mm (62.5 µg disc)	
	*C. albicans* (6 clinical)	MD and AD	2.5–331 µM, IZ: 27–34 mm (62.5 µg disc)	
	*C. parapsilosis* (2 clinical)	MD and AD	331–662 µM, IZ: 25–26 mm (62.5 µg disc)	
	*Candida zeylanoides* (clinical)	MD and AD	331 µM, IZ: 25 ± 0.4 (62.5 µg disc)	
	*Colletotrichum acutatum* (isolated from infected pomegranates)	MD	165.4 µM	
	*Coniella granati* (isolated from infected pomegranates)	MD	165.4 µM	
	*C. neoformans* ATCC 11240	MD and AD	331 µM, IZ: 25 mm (62.5 µg disc)	
	*C. neoformans* B 3501 (clinical)	MD and AD	331 µM, IZ: 24 mm (62.5 µg disc)	
	*C. neoformans* var. *grubii* H99 (clinical)	MD and AD	662 µM, IZ: 23 mm (62.5 µg disc)	
	*Cryptococcus 67* (clinical)	MD and AD	662 µM, IZ: 21 ± 0.3 mm (62.5 µg disc)	
	*Saccharomyces cerevisiae* (clinical)	MD and AD	662 µM, IZ: 23 ± 0.2 (62.5 µg disc)	
Plant Source (Part Used)				
*Excoecaria lucida* Sw (Leaves)	*C. glabrata* (B63155)	MD	128 µg/mL	[46]
	*C kefyr* (B46120)	MD	128 µg/mL	
	*C. krusei ATCC B68404*	MD	128 µg/mL	
	*C. parapsilosis* ATCC J941058	MD	128 µg/mL	
*Eugenia uniflora* (Leaves)	*C. albicans* ATCC 90028	MD	500 µg/mL	[45]
	*C. auris* ATCC CDC B11903	MD	1000 µg/mL	
	*C. glabrata* ATCC 9001	MD	125 µg/mL	
*Lafoensia pacari* (Stem bark)	*C. albicans* ATCC 10231	MD and AD	500 µg/mL, IZ: 13 mm (100 µg discs)	[49]
	*C. krusei* ATCC 6258	MD and AD	125 µg/mL, IZ: 15 mm (100 µg discs)	
	*C. parapsilosis* ATCC 22019	MD and AD	125 µg/mL, IZ: 20 mm (100 µg discs)	
	*C. tropicalis* ATCC 750	MD and AD	125 µg/mL, IZ: 13 mm (100 µg discs)	
	*S. cerevisiae* ATCC 9763	MD and AD	62 µg/mL, IZ: 17 mm (100 µg discs)	
*Klainedoxa gabonenses* (Stem bark)	*Mucor miehei*	MD and AD	31.4 µg/mL, IZ: 14 mm (20 µg discs)	[48]
	*C. albicans* (clinical)	MD and AD	30.6 µg/mL, IZ: 15 mm (20 µg discs)	

* MD: Broth Microdilution; AD: Agar Diffusion; MIC: Minimum Inhibitory Concentration; IZ: Inhibition Zone diameter. The concentrations shown on the discs correspond to the mass of pure EA.

**Table 3:** Overview of in vivo studies evaluating the antifungal efficacy and protective effect of EA in different animal models.

Fungal Pathogen	Animal Model	Dosage	Route	Duration of Dosage (Days)	Survival Rates	Key Therapeutic Outcome	Reference
*T. rubrum*	Guinea pig	4.0 and 8.0 mg/cm^2^	Topical	14	89% (8.0 mg/cm^2^) and 75% (4.0 mg/cm^2^)	Complete lesion resolution and negative mycology, demonstrating efficacy comparable to terbinafine	[66]
*C. auris* and *C. albicans*	*G. mellonella*	32 mg/kg	Intrahemocoelic	5	44%	A single pre-treatment dose of EA significantly increased survival by 44% and prolonged survival time post-infection.	[24]
*C. albicans*	*C. elegans*	4 µg/mL	Oral	6	40%	Increased nematode survival rates, confirming a protective antifungal effect in vivo.	
*C. neoformans*	Leukopenic mouse	20 and 40 mg/kg	Intraperitoneal	40	20% (20 mg/kg) and 30% (40 mg/kg)	Treatment (40 mg/kg) significantly improved survival (30% vs. 0% in controls at 40 days and reduced lung fungal burden in immunocompromised mice.	[63]
*C. albicans*	*D. melanogaster*	3.2, 6.4 and 32 µg/mL	Oral	8	45%, 33% and 34%	EA treatment provided a significant survival benefit and protection against infection without evidence of toxicity at the effective dose.	[25]

**Table 4:** Overview of advanced formulation strategies to improve the solubility, bioavailability, and efficacy of ellagic acid.

Formulation Type *	Key Findings/Improvements	Limitations/Notes	Reference
Liposomal EA (Lip-EA)	Significantly enhanced in vivo efficacy in a murine cryptococcosis model 70% survival rate (vs. 20% with fluconazole) Reduced fungal load in lungs Alleviated drug-induced toxicity	Complex and potentially costly manufacturing Stability and shelf-life concerns Study focused on intraperitoneal administration	[63]
Polymeric Hydrogel (Gellan Gum)	Effective vehicle for topical/oral application Sustained release profile demonstrated Significant reduction in *C. albicans* biofilm s and inhibition of hyphal formation High biocompatibility with human cells	Primarily suited for localized/topical therapy (e.g., oral cavity) Release kinetics and efficacy are dependent on polymer concentration	[74]
Nanoparticles (Gallium NPs coated with EA)	Demonstrated antifungal activity against *Aspergillus terreus* Represents a novel combinatorial approach (EA + metal ion)	Preliminary evidence, limited to agar diffusion assay Mechanism of action and full spectrum of activity are not yet well characterized	[100]
Cyclodextrin Complex (e.g., HP-β-CD)	Increased aqueous solubility of EA Showed efficacy in a murine model of oral candidiasis, reducing tissue invasion Effective against mature biofilms in vitro	Did not consistently improve antifungal MIC in all in vitro models The improvement in solubility does not always directly translate to a proportional increase in antimicrobial potency	[23,68]
Subcutaneous Formulations (Plain EA & EA-NPs)	Improved systemic bioavailability compared to oral administration in a toxicity model Demonstrated feasibility of parenteral delivery	Study focused on antioxidant/ nephroprotective effects, not direct antifungal efficacy Parenteral route is less desirable for chronic outpatient therapy	[30]

* Lip-EA, Liposomal Ellagic Acid; HP-β-CD, (2-Hydroxypropyl)-β-cyclodextrin; Gellan Gum (GG) was used as the polymer base for the hydrogel; NPs, Nanoparticles; EA-NPs, Ellagic Acid-loaded Nanoparticles.

## Data Availability

No new data was created or analyzed in this study. Data sharing is not applicable to this article.

## Abbreviations

The following abbreviations are used in this manuscript:

EA	Ellagic acid
WHO	World Health Organization
MIC	Minimum Inhibitory Concentration
ATCC	American Type Culture Collection
HHDP	Hexahydroxydiphenic acid
LIP-EA	Liposomal ellagic acid
AST	Aspartate aminotransferase
ALT	Alanine Aminotransferase
BUN	Blood Urea Nitrogen
CDs	Cyclodextrins
MD	Microdilution
AD	Agar Diffusion
CLSI	Clinical and Laboratory Standards Institute
EUCAST	European Committee on Antimicrobial Susceptibility Testing
CAPE	Caffeic acid
IC_50_	Inhibitory Concentration 50%
GG	Gellan gum
EA–GaNPs	Newly synthesized gallium nanoparticles coated with EA
EA/HP-β-CD	EA complexed with cyclodextrin
EA-NPs	Ellagic Acid-loaded Nanoparticles
SEDDS	Self-Emulsifying Drug Delivery Systems
NAC	non-*albicans Candida*
FICI	Fractional Inhibitory Concentration Index
EA-GaNPs	Gallium nanoparticles coated with ellagic acid
isoUro-A	Urolithin A Isomer
Uro-D	Urolithin D
Uro-E	Urolithin E
Uro-M6	Urolithin M6
Uro-C	Urolithin C
Uro-M7	Urolithin M7
Uro-A	Urolithin A
Uro-B	Urolithin B
Uro-M5	Urolithin M5
